# An Unbiased Approach to Mapping the Signaling Network of the Pseudorabies Virus US3 Protein

**DOI:** 10.3390/pathogens9110916

**Published:** 2020-11-05

**Authors:** Robert J. J. Jansens, Sandra Marmiroli, Herman W. Favoreel

**Affiliations:** 1 Department of Virology, Parasitology and Immunology, Faculty of Veterinary Medicine, Ghent University, 9820 Merelbeke, Belgium; robert.jansens@ugent.be; 2 Cellular Signaling Laboratory, Department of Surgery, Medicine, Dentistry, and Morphology, University of Modena & Reggio Emilia, 41121 Modena, Italy; sandra.marmiroli@unimore.it

**Keywords:** US3, kinase, PRV, pseudorabies virus, alphaherpesvirus, phosphoproteome, mass spectrometry

## Abstract

The US3 serine/threonine protein kinase is conserved among the alphaherpesvirus family and represents an important virulence factor. US3 plays a role in viral nuclear egress, induces dramatic alterations of the cytoskeleton, represses apoptosis, enhances gene expression and modulates the immune response. Although several substrates of US3 have been identified, an unbiased screen to identify US3 phosphorylation targets has not yet been described. Here, we perform a shotgun and phosphoproteomics analysis of cells expressing the US3 protein of pseudorabies virus (PRV) to identify US3 phosphorylation targets in an unbiased way. We identified several cellular proteins that are differentially phosphorylated upon US3 expression and validated the phosphorylation of lamin A/C at serine 404, both in US3-transfected and PRV-infected cells. These results provide new insights into the signaling network of the US3 protein kinase and may serve as a basis for future research into the role of the US3 protein in the viral replication cycle.

## 1. Introduction

Herpesviruses are among the most successful pathogens worldwide, establishing lifelong latent infections in their natural host. Their success correlates with the tight control that herpesviruses exert on different cellular pathways, sometimes by mimicking cellular proteins. One strategy to gain control over the host cell employed by herpesviruses is the expression of viral protein kinases that phosphorylate a wide range of both viral and cellular proteins [1,2]. All herpesviruses encode at least one protein kinase, while alphaherpesviruses encode two. The herpes simplex virus (HSV) and pseudorabies virus (PRV) homologues of the protein kinase conserved in all herpesviruses are called UL13. The conserved alphaherpesvirus protein kinase homologues in HSV and PRV are called US3. Although neither of these viral kinases are required for replication in cell culture, mutant viruses deficient in either kinase are severely attenuated in in vivo infection models [3,4,5,6].

The US3 protein is a multifunctional serine/threonine protein kinase. US3 expression modulates a wide array of cellular processes, including virus nuclear egress, inhibition of apoptosis, reorganization of the cytoskeleton and several immune modulators [2,7,8]. Some of these effects have been investigated at a mechanistic level, and several phosphorylation targets of US3 have been identified [2,9]. The US3 protein of HSV1 has been shown to directly phosphorylate Lamin A/C, IRF3, TSC2, Beclin1, Bad, PKA and KIF3A, in addition to several viral proteins [10,11,12,13,14,15,16,17]. The US3 protein of PRV, on the other hand, has been shown to directly phosphorylate PAK1 and PAK2 [18]. These phosphorylated proteins were identified in hypothesis-driven experiments and likely do not encompass the entire spectrum of US3 phosphorylation targets. In addition to directly phosphorylating different targets, US3 may also modulate the activity of several cellular kinases and/or phosphatases, an aspect missed when identifying direct phosphorylation targets [19,20].

A better understanding of the signaling networks affected by the US3 protein would enhance our understanding of the functions of the US3 protein, and the underlying mechanisms. Here, we used an unbiased mass spectrometry (MS) approach to identify pathways modulated by the PRV US3 protein. Mass spectrometry-based proteomics has previously been used in several studies to gain a better overview of differentially regulated or phosphorylated proteins during alphaherpesvirus infections [21,22,23,24]. By using transfected cells, we focused on proteins differentially phosphorylated by expression of US3 without the complicating background of a viral infection. Indeed, viral infection could confound findings, among others due to the expression of another viral kinase, UL13, and other viral proteins affecting the phosphorylation state of cellular proteins [5]. This approach allows us to generate a wide overview of cellular pathways affected by US3. Several differentially phosphorylated proteins involved in pathways known to be affected by US3 were identified. In addition, we were able to identify and confirm a specific serine residue in lamin A/C that is phosphorylated via US3, leading to new insights into lamin phosphorylation in herpesvirus infections [11]. Surprisingly, we also found several proteins involved in RNA processing to be differentially phosphorylated, hinting to a hitherto unknown function of US3 in mRNA processing.

## 2. Results

### 2.1. The Phosphoproteome of US3-Transfected Cells

In order to discover phosphorylation targets of the PRV US3 protein, we transfected swine testicle (ST) cells with a plasmid encoding the full length US3 protein, or a kinase inactive version of this plasmid (US3 KD) [8]. Cells were lysed at 16 hpt because the activity of US3 (anti-apoptosis and intercellular transport by TNTs) causes a higher apparent transfection efficiency compared to kinase negative US3 at later time points [25,26]. The average transfection efficiencies of US3 and US3 KD at 16 hpt were 27% and 26%, respectively (Figure S1).

**Figure 1:**
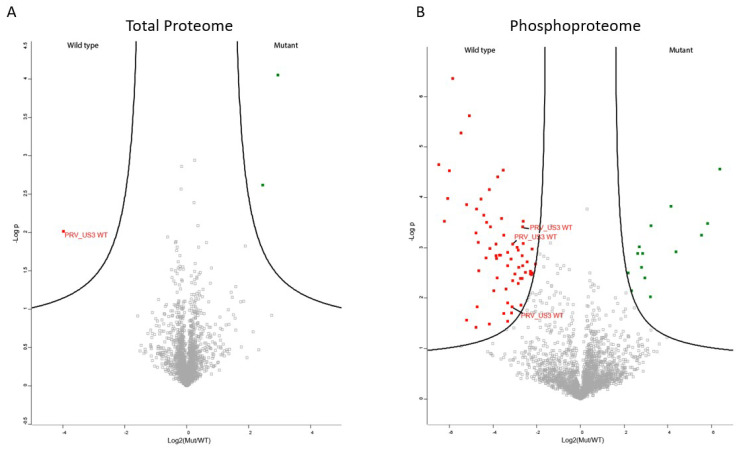
Proteome and phosphoproteome of US3-transfected cells. (**A**) Volcano plot of differential expression of proteins between ST cells expressing active US3 or kinase inactive US3. Proteins upregulated in wild type US3-expressing cells are shown in red, proteins downregulated in wild type US3-expressing cells are shown in green. (**B**) Volcano plot of differential phosphorylation of proteins between ST cells expressing active US3 or kinase negative US3. Proteins that show increased phosphorylation in wild type US3-expressing cells are shown in red, proteins that show reduced phosphorylation in wild type US3-expressing cells are shown in green.

The transfected cell lysates were used both for shotgun proteomics, determining total protein levels, and for phosphoproteomics. Since PRV US3 functions as a kinase, no major changes in total protein expression were expected. In line with this, only three proteins were found to be differentially regulated in the shotgun proteomics experiment (Figure 1A). Even though the transfection efficiencies were similar, the wild type, active version of PRV US3 showed higher expression compared to the inactive form of US3, which is in line with previous observations in our lab that kinase-active US3 appears to be more stable than its kinase negative version (unpublished results). Additionally, a component of the mitoribosome and a DNA topoimerase were found to be downregulated in cells expressing wild type US3 (Table S1).

The phosphoproteome experiment yielded a larger amount of differentially phosphorylated peptides (Figure 1B). In total, 14 cellular peptides were significantly dephosphorylated in wild type US3 transfected cells compared to kinase inactive US3 transfected cells (Table 1 and Table S1), while 64 cellular peptides showed significantly increased phosphorylation upon wild type US3 transfection compared to kinase inactive US3 transfection (Table 2 and Table S1). The list of significantly phosphorylated sites includes four sites in lamin A and C, a component of the nuclear lamina previously shown to be phosphorylated by the HSV1 homologue of US3 [11]. However, several other proteins known to be differentially phosphorylated by HSV or PRV US3, including group A PAKs, Bad, cofilin and RhoA, were not identified [8,18,20]. Our results do not indicate that these phosphorylation sites were not differentially phosphorylated, merely that these sites were not identified using our MS approach. This shows the value of using alternative methodologies to identify phosphorylation sites and indicates that the list of phosphorylated sites upon US3 expression is not exhaustive.

### 2.2. Validation of Identified S404 Phosphosite in Lamin A/C

**Figure 2:**
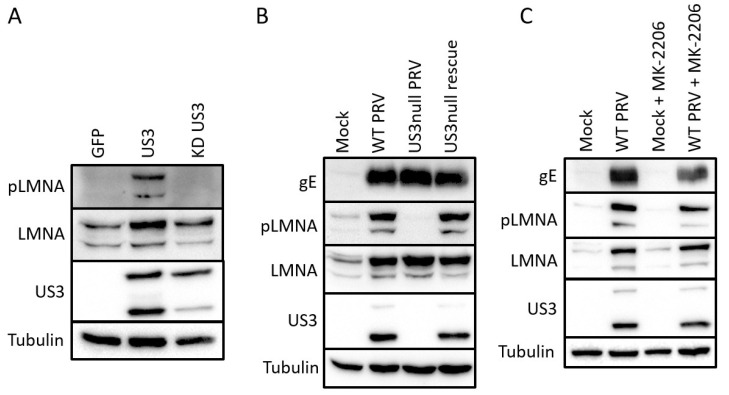
US3 triggers S404 phosphorylation of lamin A/C. (**A**) Swine testicle (ST) cells transfected for 48 h with eukaryotic expression vectors encoding GFP (pTrip), wild type US3 (pKG1) or kinase inactive US3 (pHF61). (**B**) ST cells were either mock infected, or infected with wild type (WT), US3null or US3null rescue PRV (16hpi). (**C**) ST cells were mock infected, or infected with wild type PRV. At 2 hpi, the virus inoculum was washed away and cells were treated with 5 µM of the Akt inhibitor MK-2206 for an additional 14 h.

The majority of all sites identified as differentially phosphorylated have not been functionally described before, and consequently do not have phospho-specific tools available. One exception is the S404 site of lamin A/C. This phosphosite shows an approximately 10-fold increase in phosphorylation in US3-transfected cells compared to cells transfected with kinase-inactive US3 (Table S1). Lamin A/C S404 has been previously described as a phosphorylation target of Akt and S404 phosphorylation was shown to target lamin A/C for degradation [27,28,29]. We used a S404 phospho-specific antibody to validate the mass spectrometry results. Transfection of active US3 indeed leads to an increase in p-S404 lamin A/C signal in Western blotting assays (Figure 2A). To investigate whether these results bear relevance in an infection context, Western blotting was performed in ST cells that were mock-infected or infected with wild type, US3null or a rescue virus (Figure 2B). A clear increase in p-S404 lamin A/C signal was observed in cells infected with wild type or rescue virus, while this signal was absent in cells infected with a virus that does not express US3. Total lamin A/C blotting also revealed an increase in lamin A/C levels during infection, but this effect was independent of US3 expression. An effect of PRV infection on total lamin A/C levels also appeared to be cell type dependent as lamin A/C levels were reduced upon infection in rabbit or swine kidney cells (RK-13 and SK cells) (data not shown). S404 of lamin A/C was initially described as a phosphorylation target of Akt [27,28,29]. In order to find out whether the US3 triggered phosphorylation of lamin A/C occurs through Akt, we infected cells with PRV in the absence or presence of 5 µM of the Akt inhibitor MK-2206. MK-2206 treatment did not affect phosphorylation of S404 in Lamin A/C, in line with a previous report of HSV1 US3 acting as an Akt mimic, rather than as an Akt-triggering viral protein (Figure 2C) [12].

### 2.3. Gene Enrichment Analysis

**Figure 3:**
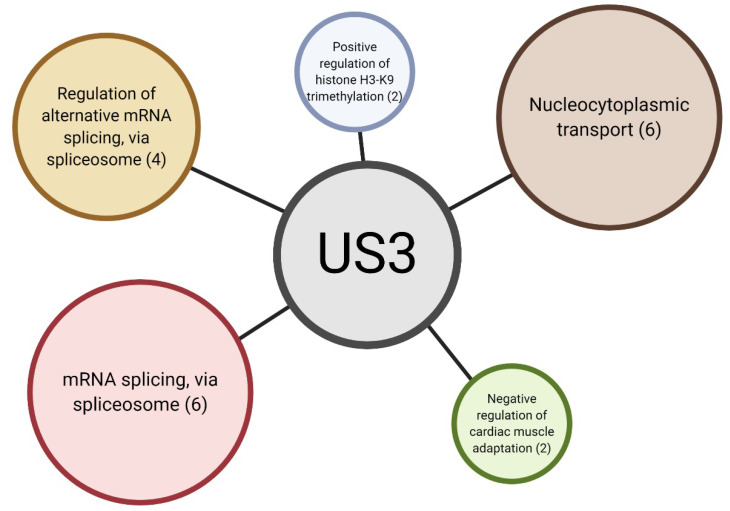
Overview of significantly enriched gene ontologies in differentially phosphorylated proteins. Each circle represents a significantly enriched gene ontology. The number between brackets is the number of proteins present in this ontology. Figure created via BioRender.com.

In order to identify specific pathways affected by US3 expression, we performed gene ontology (GO) enrichment analysis of differentially phosphorylated proteins (Figure 3 and Table S2). Surprisingly, we found biological processes related to RNA processing to be substantially enriched. Apart from promoting transcriptional activity by phosphorylation of HDAC proteins, US3 has no known functions in RNA processing. Biological processes related to “nucleocytoplasmic transport” were also overrepresented in differentially phosphorylation sites (Table S2). It is therefore possible that some of the functions of the US3 protein are performed by shuttling proteins between the nucleus and the cytoplasm.

“Negative regulation of cardiac muscle adaptation” and “positive regulation of histone H3-K9 trimethylation” were both found to also be enriched, however these ontologies are poorly annotated and each contain only two or three annotated proteins in the porcine database (Table S2). It can therefore not be excluded at this point that these ontologies may possibly represent false positives.

## 3. Discussion

The US3 protein is an important virulence factor in alphaherpesviruses and fulfills a wide array of functions in virus-host interactions of both HSV and PRV. Here, we used a mass spectrometry approach to identify proteins differentially phosphorylated upon expression of the US3 protein versus expression of a kinase-inactive US3 mutant. In total we identified 78 differentially phosphorylated phosphosites in cells transfected with wild type US3 versus cells transfected with kinase inactive US3, of which 64 were phosphorylated and 14 were dephosphorylated. These may include proteins directly phosphorylated by US3 and/or phosphosites targeted by cellular kinases or phosphatases that are activated or inactivated by US3 expression. We identified and confirmed S404 as a phosphorylation site in lamin A/C in both US3-transfected and PRV-infected cells. S404 of lamin A/C was previously shown to be phosphorylated by the cellular kinase Akt, further pointing towards US3 as a functional mimic of Akt [28,30]. US3-mediated phosphorylation of S404 lamin A/C in PRV-infected cells does not depend on Akt, as phosphorylation still occurs in the presence of an Akt inhibitor. This is in line with a study on HSV1 US3, where it was shown that the US3-dependent phosphorylation of the Akt target 4E-BP1 does not require Akt activity [12]. Akt phosphorylation of lamin A/C induces lamin degradation and an alternative morphology of the nuclear lamina, resembling the phenotype observed in Emery–Dreifuss muscular dystrophy cells [27,28,29]. Although no reduction in total lamin A/C levels was observed upon PRV infection in ST cells, a substantial reorganization of the nuclear lamina upon herpesvirus infections has been previously described [11,31,32].

Apart from lamin A/C, several promising novel phosphorylation targets were identified. Although these phosphosites have not been functionally characterized before, they might represent new research avenues, leading to a better understanding of the functions of US3. One of the most striking effects of the US3 protein is the dramatic reorganization of the actin cytoskeleton, involving the breakdown of actin stress fibers and the formation of tunneling nanotubes [7,26]. These changes were shown to depend on dephosphorylation of cofilin at position S3 [33]. Interestingly, one of the significantly phosphorylated proteins identified in our screen was testis-specific protein kinase 2 (TESK2). TESK2 belongs to the TESK/LIMK family of protein kinases, and has been shown to phosphorylate cofilin at position S3 [34,35]. Although regulation of TESK2 by phosphorylation has not been described, the activity of the related LIMK protein is regulated by phosphorylation [36]. Although speculative at this point, this may indicate that US3-induced phosphorylation of TESK2 may affect TESK2 activity, thereby modulating S3 phosphorylation of cofilin. Another differentially phosphorylated protein potentially involved in the cytoskeletal rearrangements induced by the US3 protein is ENAH. ENAH, a member of the Ena/VASP family of actin regulators, promotes the formation of filopodia [37]. Although the activity of ENAH has been previously shown to be regulated by phosphorylation [38,39], the effect of the phosphosite identified in the current screen is unknown.

Tunneling nanotubes induced by US3 are remarkably stable compared to endogenous TNTs described in literature [26]. We previously showed that they contain microtubules with post-translational modifications associated with stability, and that the contact area with connected cells is enriched in cadherins and beta-catenin [26]. Some of the proteins identified in the current phosphoproteomics screen might shed light on the mechanism underlying this TNT stabilization. Two proteins involved in the regulation of microtubule stability were found to be differentially phosphorylated. The microtubule-associated protein 1B (MAP1B) regulates microtubule dynamics and is regulated by phosphorylation [40,41,42]. Interestingly, the phosphosite that was found to be dephosphorylated in the current screen was previously found to be dephosphorylated in a phosphoproteomics screen investigating the effects of a combined inhibition of ROCK1 and epidermal growth factor [43]. ROCK1 inhibition leads to the formation of cell protrusions resembling US3-induced TNTs [7], pointing towards a possible function of S891 MAP1B dephosphorylation in TNT formation. CLASP1, another microtubule regulator, was found to be significantly phosphorylated and has previously been described to be important in US3-mediated microtubule stabilization [44]. Although the phosphosite identified in the current screen has not been characterised, it was previously identified in a screen identifying phosphorylation targets of AMP-activated protein kinase (AMPK), together with several other proteins involved in cell motility and invasion [45]. AMPK activation has previously been shown to induce stress fiber disassembly, a phenotype also observed upon US3 expression [46,47]. Finally, phosphorylation of cadherin-6, also known as K-cadherin, could also be involved in the stability of US3-induced TNTs. K-cadherin is phosphorylated in its C-terminal intracellular domain. Phosphorylation of E-cadherin in its intracellular domain enhances its association with beta-catenin and strengthens cell–cell adhesion [48,49]. Although K-cadherin phosphorylation has not been functionally described before, it is possible that K-cadherin is regulated similarly and that this may contribute to stabilization of US3-induced TNTs.

One of the most surprising findings of the current phosphoproteome screen was the large number of proteins involved in RNA processing. Gene ontology enrichment analysis showed a marked enrichment of phosphorylated proteins involved in mRNA processing. US3 has previously been shown to phosphorylate histone deacetylases (HDAC), leading to increased reporter gene expression [50,51], but the gene ontologies enriched in proteins phosphorylated by US3 are mostly related to RNA splicing. US3 is not known to affect splicing, indicating that this might be a novel function of US3.

Despite the large amount of promising new phosphosites identified in the current screen, most of the proteins that were demonstrated before to be phosphorylated by US3 were not identified. The absence of these phosphosites does not contradict previous findings. Rather, it shows that the list of US3 phosphorylation targets is not complete. While most known proteins phosphorylated by US3 were identified via hypothesis-driven experiments, our approach does not rely on assumptions or prior knowledge. Although this allows the identification of uncharacterized phosphosites, there is no selection for biologically functional sites. In order to draw conclusions on the effect of these sites, extensive validation will be required.

In conclusion, we identified a wide range of novel phosphosites affected by PRV US3 expression. These include several phosphorylated residues of lamin A/C, a known US3 substrate, of which S404 phosphorylation was validated using a phospho-specific antibody. Enrichment analysis of significantly phosphorylated proteins revealed a remarkable number of proteins involved in mRNA processing. Our results provide several research avenues that can lead to a better understanding of the functions of the US3 protein in the PRV replication cycle, as well as of the regulation of cellular proteins via phosphorylation.

## 4. Materials and Methods

### 4.1. Cells, Inhibitors and Viruses

Swine testicle (ST) cells were cultured in MEM (Gibco) supplemented with 10% fetal calf serum (FCS), 1 mM sodium pyruvate, 10^5^ U/L penicillin, 100 mg/L streptomycin and 50 mg/L gentamycin. Confluent cells were infected at an MOI of 10 and lysed at 16 hpi. Wild type NIA3, an isogenic US3null mutant and the corresponding rescue virus were previously described and were kindly donated by the ID-DLO (The Netherlands) [52,53]. The Akt inhibitor MK-2206 was purchased from Selleckchem. Cells were infected for 2 h after which the initial inoculum was washed away and 5 µM of MK-2206 in complete medium was added, in order to prevent the inhibitor from interfering with viral entry.

### 4.2. Transfection

Cells were transfected with 2250 ng of plasmid DNA for each well of a 6 well plate using JetPrime (poly-plus) according to the manufacturer’s instructions. The plasmids encoding full length wild type NIA3 US3 protein (pKG1) and full length kinase inactive NIA3 US3 protein with a K138Q mutation (pHF61) were described previously [8,25]. These plasmids allow the expression of both the short and long isoform of the US3 proteins, as is the case during PRV infection. The pTrip plasmid encoding eGFP was a kind gift of B. Verhasselt (Ghent University, Ghent, Belgium). Cells intended for mass spectrometry were lysed at 16 h post transfection (hpt) to prevent large differences in US3 expression between the active and the kinase inactive US3 plasmid. Three replicates were performed. Cells transfected in parallel were used to determine the transfection efficiency by immunofluorescence followed by flow cytometry. Transfection efficiencies of both samples were similar in each of the replicates.

### 4.3. Flow Cytometry

Cells transfected with US3 or kinase negative US3 were collected by trypsinization. After washing the cells with PBS, the cells were fixed using 3% paraformaldehyde and permeabilized using 0.1% saponin (Sigma Aldrich, St. Louis, MO, USA). Cells were incubated with a 1/100 dilution of a mouse anti-US3 antibody in PBS with 0.1% saponin. The anti-US3 antibody was a kind gift of Leigh Anne Olsen and Lynn Enquist (Princeton University, Princeton, NJ, USA). Following three washing steps in PBS, cells were incubated with a 1/200 dilution of goat anti-mouse AF647 antibody (ThermoFisher, Waltham, MA, USA). After three additional washing steps the cells were analyzed using a BD FACSAria III Cell Sorter (BD Biosciences, Franklin Lakes, NJ, USA).

### 4.4. Mass Spectrometry Sample Preparation

Cells were lysed in a urea lysis buffer containing 9 M urea, 20 mM HEPES pH 8.0 and PhosSTOP phosphatase inhibitor cocktail (Roche, Basel, Switzerland), 1 tablet/10 mL buffer). The samples were sonicated with 3 pulses of 15 s at an amplitude of 20% using a 3 mm probe, with incubation on ice for 1 min between pulses. After centrifugation for 15 min at 20,000× *g* at room temperature to remove insoluble components, proteins were reduced by addition of 5 mM DTT and incubation for 30 min at 55 °C and then alkylated by addition of 10 mM iodoacetamide and incubation for 15 min at room temperature in the dark. The protein concentration was measured using a Bradford assay (Bio-rad) and from each sample 2 mg protein was used to continue the protocol. Samples were further diluted with 20 mM HEPES pH 8.0 to a final urea concentration of 4 M and proteins were digested with 8 µg LysC (Wako, Osaka, Japan) (1/250, *w*/*w*) for 4 h at 37 °C. Samples were again diluted to 2 M urea and digested with 20 µg trypsin (Promega) (1/100, *w*/*w*) overnight at 37 °C. The resulting peptide mixture was acidified by addition of 1% trifluoroacetic acid (TFA) and after 15 min incubation on ice, samples were centrifuged for 15 min at 1780× *g* at room temperature to remove insoluble components. Next, peptides were purified on SampliQ SPE C18 cartridges (500 mg, Agilent, Santa Clara, USA). Columns were first washed with 5 mL 100% acetonitrile (ACN) and pre-equilibrated with 15 mL of solvent A (0.1% TFA in water/ACN (98:2, *v*/*v*)) before samples were loaded on the column. After peptide binding, the column was washed again with 5 mL of solvent A and peptides were eluted twice with 700 µL elution buffer (0.1% TFA in water/ACN (20:80, *v*/*v*)). The eluted peptides were divided in two parts: 100 µL was dried completely in a speedvac vacuum concentrator for shotgun analysis, while the remainder was used for phosphopeptide enrichment. Phosphopeptides were enriched with MagReSyn^®^ Ti-IMAC beads following the protocol according to the manufacturer’s instructions with slight modifications. Briefly, 200 µl MagReSyn^®^ Ti-IMAC beads (per sample) were washed twice with 70% EtOH, once with 1% NH_4_OH and three times with a mixture of water/ACN/TFA (14:80:6, *v*/*v*/*v*). Next, the digested sample was incubated with the washed beads for 30 min at room temperature, the beads were washed once with a mixture of water/ACN/TFA (14:80:6, *v*/*v*/*v*) and three times with a mixture of water/ACN/TFA (19:80:1, *v*/*v*/*v*). Phosphopeptides were eluted from the beads by adding three times 80 µl 1% NH_4_OH. In total, 60 µL 10% formic acid (FA) was added to the combined eluate and the samples were dried completely in a speedvac vacuum concentrator.

### 4.5. LC-MS/MS Analysis

Purified peptides for shotgun analysis were re-dissolved in 100 µL solvent A and the peptide concentration was determined on a Lunatic spectrophotometer (Unchained Labs) [54]. In total, 2 µg of each sample was injected for LC-MS/MS analysis on an Ultimate 3000 RSLCnano system (Thermo) in-line connected to a Q Exactive HF mass spectrometer (Thermo) equipped with a Nanospray Flex Ion source (Thermo). Peptides resulting from phosphopeptide enrichment were re-dissolved in 20 µl solvent A of which 10 µL was injected for LC-MS/MS analysis on the same system. Trapping of peptides was performed at 10 μL/min for 4 min in solvent A on a 20 mm trapping column (made in-house, 100 μm internal diameter (I.D.), 5 μm beads, C18 Reprosil-HD, Dr. Maisch, Ammerbuch, Germany) and the sample was loaded on an analytical column packed in the needle (made in-house, 75 μm I.D. × 400 mm, 1.9 μm beads C18 Reprosil-HD, Dr. Maisch). Peptides were eluted by a non-linear increase from 2 to 56% MS solvent B (0.1% FA in water/ACN (2:8, *v*/*v*)) over 145 min at a constant flow rate of 250 nl/min and a constant temperature of 50 °C (CoControl 3.3.05, Sonation), followed by a 15-min wash reaching 99% MS solvent B and re-equilibration with MS solvent A (0.1% FA in water/ACN (2:8, *v*/*v*)). The mass spectrometer was operated in data-dependent mode, automatically switching between MS and MS/MS acquisition for the 16 most abundant ion peaks per MS spectrum. Full-scan MS spectra (375–1500 m/z) were acquired at a resolution of 60,000 in the orbitrap analyser after accumulation to a target value of 3,000,000. The 16 most intense ions above a threshold value of 22,000 (shotgun) or 13,000 (phospho) were isolated (window of 1.5 Th) for fragmentation at a normalized collision energy of 28% after filling the trap at a target value of 100,000 for maximum 45 ms (shotgun) or 80 ms (phospho). MS/MS spectra (200–2000 m/z) were acquired at a resolution of 15,000 in the orbitrap analyser. The S-lens RF level was set at 55 and we excluded precursor ions with single, unassigned and >6 charge states from fragmentation selection. QCloud was used to control instrument longitudinal performance during the project [55].

### 4.6. Data Analysis

Analysis of the shotgun and phosphoproteomics data was performed in two separate MaxQuant searches (version 1.5.6.5) with identical, mainly default search settings including a false discovery rate set at 1% on PSM, peptide and protein level. Spectra were searched against the *Sus scrofa* proteins in the Uniprot/Swiss-Prot database (database release version of November 2016 containing 26,101 pig protein sequences, downloaded from http://www.uniprot.org) supplemented with the sequences of the kinase active/inactive US3 Pseudorabies virus (NIA3 strain). The mass tolerance for precursor and fragment ions was set to 4.5 and 20 ppm, respectively, during the main search. Enzyme specificity was set as C-terminal to arginine and lysine, also allowing cleavage at proline bonds with a maximum of two missed cleavages. Variable modifications were set to oxidation of methionine residues, acetylation of protein N-termini and phosphorylation of serine, threonine or tyrosine residues, while carbamidomethylation of cysteine residues was set as fixed modification. Matching between runs was enabled with a matching time window of 1 min and an alignment time window of 20 min. Only proteins with at least one unique or razor peptide were retained leading to the identification of 4743 proteins in the shotgun samples and 5583 phosphorylation sites in the phosphopeptide enriched samples. Proteins identified in the shotgun samples were quantified by the MaxLFQ algorithm integrated in the MaxQuant software [56]. A minimum ratio count of two unique or razor peptides was required for quantification. Further data analysis was performed with the Perseus software (version 1.5.5.3) after loading the proteingroups file from MaxQuant. Reverse database hits and proteins that were only identified by site were removed, LFQ intensities were log2 transformed and replicate samples were grouped. Proteins with less than three valid values in at least one group were removed and missing values were imputed from a normal distribution around the detection limit leading to a list of 3109 quantified proteins that was used for further data analysis. Then, a t-test was performed (FDR = 0.05 and s0 = 1) to compare wild type and mutant samples and a volcano plot was generated. Three proteins were found to be significantly regulated. For further analysis of the phosphoproteomics data, the phospho(STY)sites file was loaded in the Perseus software (version 1.5.5.3). Reverse hits were removed, the site table was expanded and the intensity values were log2 transformed. Replicate samples were grouped, phosphosites with less than three valid values in at least one group were removed and missing values were imputed from a normal distribution around the detection limit leading to a list of 2971 quantified phosphopeptides that was used for further data analysis. Then, a t-test was performed (FDR = 0.05 and s0 = 1) to compare control and KO samples and a volcano plot was generated. In total, 81 phosphopeptides were significantly regulated.

The mass spectrometry proteomics data have been deposited to the ProteomeXchange Consortium via the PRIDE [57] partner repository with the dataset identifier PXD021751.

### 4.7. Western Blotting

Cells were lysed at 48 hpt or 16 hpi in RIPA buffer (Abcam, Cambridge, UK) with cOmplete mini EDTA free protease inhibitor cocktail (Roche, Basel, Switzerland ) and PhosStop (Roche). Cell lysates were separated on a 10% polyacrylamide gel, followed by blotting on PVDF membrane (GE healthcare, Piscataway, NJ, USA). Regular blots were blocked in 5% nonfat milk diluted in 0.1% Tween-20 in PBS for 1 h at room temperature. When using phosphorylated protein-specific antibodies, 5% bovine serum albumin (MP Biomedicals, Santa Ana, USA) diluted in 0.1% Tween-20 in PBS was used for blocking. Primary antibodies were incubated overnight at 4 °C (Table 3). Following 3 consecutive 5 min washes in PBS-T, the membranes were incubated with the secondary antibody for 1h at room temperature. Following 3 more 5 min washes, the blots were detected using Pierce enhanced chemiluminescence (ECL) substrate (ThermoFisher, Waltham, MA, USA), ECL Plus substrate (GE Healthcare, Piscataway, NJ, USA), or SuperSignal West Femto maximum sensitivity substrate (ThermoFisher, Waltham, MA, USA) on a ChemiDoc MP imaging device (Bio-Rad, Hercules, CA, USA).

### 4.8. Gene Ontology Enrichment Analysis

The enrichment of “biological process” gene ontologies of the differentially phosphorylated proteins was analyzed using the PANTHER overrepresentation test against the Sus Scrofa database (GO Ontology database DOI: 10.5281/zenodo.3873405 Released 2020-06-01). Fisher’s Exact test was used with a 0.05% false discovery rate correction.

## Figures and Tables

**Table 1:** Top five dephosphorylated proteins in wild type US3 transfected cells compared to kinase inactive US3 transfected cells.

Gene Name	Protein Name	Site	Log2(KD/WT)	-Log2(*p*-Value)
CDS2	Phosphatidate cytidylyltransferase	S33	6.38	4.56
BCKDHA	2-oxoisovalerate dehydrogenase alpha	S313	5.83	3.48
BCKDHA	2-oxoisovalerate dehydrogenase alpha	S303	5.54	3.25
PPP6R1	S/T-protein phosphatase 6 subunit 1	S531	4.39	2.92
WDR20	WD repeat-containing protein 20	S348	4.16	3.83

**Table 2:** Top 10 Phosphorylated proteins in US3 transfected cells compared to kinase negative US3 transfected cells.

Gene Name	Protein Name	Site	Log2(KD/WT)	-Log2(*p*-Value)
TSSC1	EARP and GARP complex interacting protein 1	S320	−6.50	4.65
RAB11FIP5	Rab11 family-interacting protein 5	T162	−6.24	3.53
RAB11FIP5	Rab11 family-interacting protein 5	S164	−6.09	3.98
TOMM70	Mitochondrial import receptor subunit TOM70	S97	−6.01	4.52
TESK2	Dual specificity testis-specific protein kinase 2	S8	−5.85	6.37
SZRD1	SUZ domain-containing protein 1	S17	−5.48	2.28
DDX17	Probable ATP-dependent RNA helicase DDX17	S575	−5.23	3.85
LMNA	Prelamin-A/C	S12	−5.21	1.56
SZRD1	SUZ domain-containing protein 1	S19	−5.09	5.62
PALMD	Palmdelphin	T255	−4.78	3.29

**Table 3:** Primary antibodies used for Western blotting.

Target	Concentration	Source
Alpha-tubulin	1/1000	ab40742, Abcam
LMNA	1/500	ab208798, Abcam
p-S404 LMNA	1/1000	[27]
US3	1/100	Leigh Anne Olsen and Lynn Enquist, [4] Princeton University, Princeton, USA

## Supplementary Materials

The following are available online at https://www.mdpi.com/2076-0817/9/11/916/s1, Table S1: Overview of shotgun and phospho- mass spectrometry data, Table S2: significantly enriched GO terms. Figure S1: Flow cytometry analysis of transfection efficiency of samples used for mass spectrometry.
