# Supplementary material for: An Unbiased Approach to Mapping the Signaling Network of the Pseudorabies Virus US3 Protein
Source: Pathogens. 2020 Nov 5;9(11):916. doi: 10.3390/pathogens9110916 (PMC7694389; doi:10.3390/pathogens9110916)
Supplement: Supplementary file 1 [file pathogens-09-00916-s001.zip › Table S2.docx]

| GO biological process complete | #Reference | #Identified | expected | Fold Enrichment | raw P value | FDR |
| --- | --- | --- | --- | --- | --- | --- |
| mRNA splicing, via spliceosome | 221 | 6 | .56 | 10.63 | 2.41E-05 | 3.13E-02 |
| - mRNA processing | 317 | 7 | .81 | 8.65 | 1.79E-05 | 2.55E-02 |
| -- mRNA metabolic process | 439 | 8 | 1.12 | 7.14 | 1.68E-05 | 2.67E-02 |
| ---RNA metabolic process | 1176 | 13 | 3.00 | 4.33 | 7.04E-06 | 2.51E-02 |
| ----nucleic acid metabolic process | 1660 | 15 | 4.24 | 3.54 | 1.36E-05 | 2.43E-02 |
| -----nucleobase-containing compound metabolic process | 2058 | 16 | 5.26 | 3.04 | 4.17E-05 | 3.50E-02 |
| ------heterocycle metabolic process | 2167 | 16 | 5.53 | 2.89 | 7.73E-05 | 4.80E-02 |
| --RNA processing | 708 | 11 | 1.81 | 6.08 | 1.70E-06 | 2.42E-02 |
| ---gene expression | 1752 | 16 | 4.47 | 3.58 | 5.66E-06 | 2.70E-02 |
| -RNA splicing, via transesterification reactions with bulged adenosine as --nucleophile | 221 | 6 | .56 | 10.63 | 2.41E-05 | 2.87E-02 |
| ---RNA splicing, via transesterification reactions | 221 | 6 | .56 | 10.63 | 2.41E-05 | 2.65E-02 |
| ----RNA splicing | 258 | 6 | .66 | 9.11 | 5.61E-05 | 4.00E-02 |
| nucleocytoplasmic transport | 189 | 6 | .48 | 12.43 | 1.02E-05 | 2.92E-02 |
| -nuclear transport | 189 | 6 | .48 | 12.43 | 1.02E-05 | 2.43E-02 |
| --establishment of localization in cell | 1330 | 13 | 3.40 | 3.83 | 2.58E-05 | 2.63E-02 |
| regulation of alternative mRNA splicing, via spliceosome | 72 | 4 | .18 | 21.75 | 4.24E-05 | 3.36E-02 |
| -regulation of mRNA splicing, via spliceosome | 110 | 5 | .28 | 17.80 | 1.11E-05 | 2.27E-02 |
| --regulation of mRNA processing | 139 | 5 | .35 | 14.09 | 3.30E-05 | 3.14E-02 |
| ---regulation of mRNA metabolic process | 224 | 7 | .57 | 12.24 | 1.96E-06 | 1.40E-02 |
| --regulation of RNA splicing | 147 | 5 | .38 | 13.32 | 4.28E-05 | 3.21E-02 |
| negative regulation of cardiac muscle hypertrophy in response to stress | 3 | 2 | .01 | > 100 | 6.35E-05 | 4.31E-02 |
| -negative regulation of cardiac muscle adaptation | 3 | 2 | .01 | > 100 | 6.35E-05 | 4.12E-02 |
| positive regulation of histone H3-K9 trimethylation | 2 | 2 | .01 | > 100 | 3.81E-05 | 3.40E-02 |
