# Supplementary figures and images for: An Unbiased Approach to Mapping the Signaling Network of the Pseudorabies Virus US3 Protein
Source: Pathogens. 2020 Nov 5;9(11):916. doi: 10.3390/pathogens9110916 (PMC7694389; doi:10.3390/pathogens9110916)

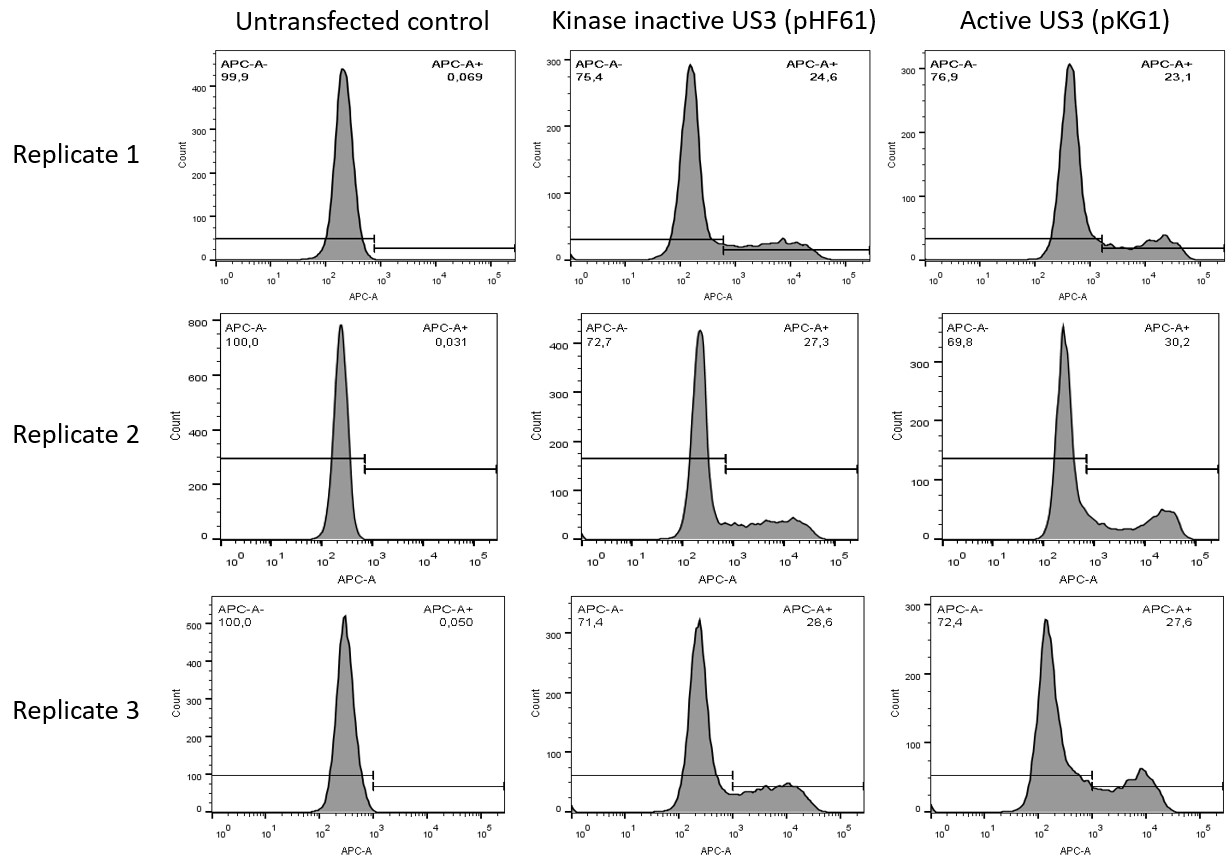

Supplement: Supplementary file 1 [file pathogens-09-00916-s001.zip › Supplementary Figure 1.jpg]
